# Cognitive and Clinical‐Functional Outcomes in Elderly People with Post‐COVID Syndrome: Data from a Brazilian Cohort

**DOI:** 10.1002/alz70857_107555

**Published:** 2025-12-25

**Authors:** Fabiana Carla Matos da Cunha Cintra, Marco Túlio Cintra, Henrique Dias Furtado De Souza, Luciano Pimenta Júnior, Gabriel de Faria Chaimowicz, Marcus Vinicius Miranda Macedo, Iza de Faria Fortini, Bernardo de Mattos Viana, Maria Aparecida Camargos Bicalho

**Affiliations:** ^1^ Federal University of Minas Gerais, Belo Horizonte, Minas Gerais, Brazil; ^2^ UFMG, Belo Horizonte, Minas Gerais, Brazil; ^3^ National Institute of Science and Technology Neurotec R (INCT‐MM), Faculdade de Medicina, Universidade Federal de Minas Gerais, Belo Horizonte, Brazil; ^4^ Neurotec R National Institute of Science and Technology (INCT‐Neurotec R), Faculty of Medicine, Universidade Federal de Minas Gerais (UFMG), Belo Horizonte, Minas Gerais, Brazil; ^5^ Molecular Medicine Postgraduate Program, School of Medicine, Universidade Federal de Minas Gerais (UFMG), Belo Horizonte, Minas Gerais, Brazil; ^6^ Older Adult's Psychiatry and Psychology Extension Program (PROEPSI), School of Medicine, Universidade Federal de Minas Gerais (UFMG), Belo Horizonte, Minas Gerais, Brazil; ^7^ Universidade Federal de Minas Gerais, Belo Horizonte, Minas Gerais, Brazil; ^8^ Cog‐Aging Research Group, Universidade Federal de Minas Gerais (UFMG), Belo Horizonte, Minas Gerais, Brazil; ^9^ Department of Clinical Medicine, Faculty of Medicine, Universidade Federal de Minas Gerais (UFMG), Belo Horizonte, Minas Gerais, Brazil; ^10^ Geriatrics and Gerontology Center Clinical Hospital of Universidade Federal de Minas Gerais, Belo Horizonte, Minas Gerais, Brazil; ^11^ National Institute of Science and Technology Neurotec R (INCT‐MM), Belo Horizonte, Minas Gerais, Brazil

## Abstract

**Background:**

Post‐COVID Syndrome (PCS) is characterized by the persistence or emergence of new symptoms for more than 12 weeks after microbiological cure of Sars‐CoV‐2 infection. The objective of this study was to identify clinical, cognitive and functional changes presented by elderly people with (PCS).

**Method:**

This is a cross‐sectional study nested in a prospective cohort that recruited elderly individuals and their respective caregivers after a laboratory‐confirmed diagnosis of COVID‐19, from 2021 to 2024. All participants signed the Informed Consent Form (CAAE: 31799420.6.0000.5149). Sociodemographic variables were assessed and the following tests were applied: The Mattis Dementia Rating Scale (DRS), Alzheimer's Disease Cooperative Study‐ activities of daily living (ADCS‐ADL), Post‐Traumatic Stress Disorder Checklist ‐ Civilian Version (PCL‐C), DSM‐5 criteria for major depressive disorder, Clinical‐Functional Vulnerability Index (IVCF‐20), Fatigue Severity Scale (FSS), mMRC dyspnea scale and SARC‐F+CC. Descriptive statistical analysis was used.

**Result:**

The sample consisted of 80 participants. The mean time between initial infection and the 1st evaluation was 10.09 ± 5.97 months (Min.: 2‐Max.: 24). 51.25% of the participants were female, while 57.50% were hospitalized, with a mean age of 73.91 ± 10.20 years; 8.03 ± 3.24 years of schooling, 6.54 ± 3.24 medications in use, Charlson Comorbidity Index of 1.79 ± 1.80 and 3.39 ± 1.63 doses of vaccine for Covid‐19 at the time of diagnosis. 70.87% reported memory difficulty after Covid‐19 and regarding the diagnosis, 15% had dementia; 62.5% mild cognitive impairment and 22.5% preserved cognition. The prevalence of Frailty was 37.50% and Functional Disability was 73.33%. Fatigue was present in 46.25% of participants, dyspnea in 88.75%, Sarcopenia in 26.25%, 11.39% screened positive for Post‐Traumatic Stress Disorder and 11.25% for major depression.

**Conclusion:**

The results of this study indicate a high prevalence of Cognitive and Functional Disability, Frailty, Fatigue, and Dyspnea after the initial diagnosis of Covid‐19. Accurate and early identification of CPS symptoms in the elderly is important to provide adequate treatment, prevent long‐term complications and improve the quality of life of this population.

